# Validation of an Improved Computer-Assisted Technique for Mining Free-Text Electronic Medical Records

**DOI:** 10.2196/medinform.7123

**Published:** 2017-06-29

**Authors:** Marco Duz, John F Marshall, Tim Parkin

**Affiliations:** ^1^ School of Veterinary Medicine and Science University of Nottingham Loughborough United Kingdom; ^2^ School of Veterinary Medicine College of Medical, Veterinary and Life Sciences University of Glasgow Glasgow United Kingdom

**Keywords:** text mining, data mining, electronic medical record, validation studies

## Abstract

**Background:**

The use of electronic medical records (EMRs) offers opportunity for clinical epidemiological research. With large EMR databases, automated analysis processes are necessary but require thorough validation before they can be routinely used.

**Objective:**

The aim of this study was to validate a computer-assisted technique using commercially available content analysis software (SimStat-WordStat v.6 (SS/WS), Provalis Research) for mining free-text EMRs.

**Methods:**

The dataset used for the validation process included life-long EMRs from 335 patients (17,563 rows of data), selected at random from a larger dataset (141,543 patients, ~2.6 million rows of data) and obtained from 10 equine veterinary practices in the United Kingdom. The ability of the computer-assisted technique to detect rows of data (cases) of colic, renal failure, right dorsal colitis, and non-steroidal anti-inflammatory drug (NSAID) use in the population was compared with manual classification. The first step of the computer-assisted analysis process was the definition of inclusion dictionaries to identify cases, including terms identifying a condition of interest. Words in inclusion dictionaries were selected from the list of all words in the dataset obtained in SS/WS. The second step consisted of defining an exclusion dictionary, including combinations of words to remove cases erroneously classified by the inclusion dictionary alone. The third step was the definition of a reinclusion dictionary to reinclude cases that had been erroneously classified by the exclusion dictionary. Finally, cases obtained by the exclusion dictionary were removed from cases obtained by the inclusion dictionary, and cases from the reinclusion dictionary were subsequently reincluded using Rv3.0.2 (R Foundation for Statistical Computing, Vienna, Austria). Manual analysis was performed as a separate process by a single experienced clinician reading through the dataset once and classifying each row of data based on the interpretation of the free-text notes. Validation was performed by comparison of the computer-assisted method with manual analysis, which was used as the gold standard. Sensitivity, specificity, negative predictive values (NPVs), positive predictive values (PPVs), and F values of the computer-assisted process were calculated by comparing them with the manual classification.

**Results:**

Lowest sensitivity, specificity, PPVs, NPVs, and F values were 99.82% (1128/1130), 99.88% (16410/16429), 94.6% (223/239), 100.00% (16410/16412), and 99.0% (100×2×0.983×0.998/[0.983+0.998]), respectively. The computer-assisted process required few seconds to run, although an estimated 30 h were required for dictionary creation. Manual classification required approximately 80 man-hours.

**Conclusions:**

The critical step in this work is the creation of accurate and inclusive dictionaries to ensure that no potential cases are missed. It is significantly easier to remove false positive terms from a SS/WS selected subset of a large database than search that original database for potential false negatives. The benefits of using this method are proportional to the size of the dataset to be analyzed.

## Introduction

Exploitation of clinical information in electronic medical records (EMRs) has the potential to revolutionize medical research. Even though time consuming, the assumed gold standard for analysis of medical data consists of manual evaluation, and this is what automated analysis tools should be validated against [1]. Data in an EMR could be used to perform epidemiological studies to support updated disease registries, drug safety surveillance, clinical trials, and health audits [2]. One of the aims of the Department of Health in the United Kingdom is to achieve a paperless National Health Service (NHS) by 2018 [3]. This plan would result in conversion of the medical records of the whole British population to a digital format, and therefore, potentially make it available for epidemiological research. Efficiency of algorithms for anonymization of an EMR have also been thoroughly evaluated [4,5], so considerations related to protection of patient’s confidentiality are unlikely to pose a limitation to these studies. Many EMR management systems are currently in use in medical practice, and this poses a challenge to research as these systems store data using different formats. However, while these systems present substantial technical differences, at a minimum, data is generally stored with a combination of structured data (patient ID, location, and date) and unstructured free-text clinical notes, often including further information such as diagnostic imaging, laboratory reports, and billing information where applicable. Data stored in these systems could be used for epidemiologic research using methodologies that are independent from the system used [2]. Coding of medical records is often implemented to support a clear classification of clinical cases but limits a clinician’s freedom of expression and often does not entirely suit all details of the clinical case and relies on clinicians to use the coding system correctly [6].

Text mining techniques have been developed over the past 40 years [7-10]; they use tools compatible with both structured and unstructured data. Text mining aims to extract information of interest from a dataset and transform this information into an understandable structure for future use [11]. A recent study compared the accuracy of information extraction between the main text mining tools currently available for the purpose of case-detection for named clinical conditions [2]. Commonly used methods include rule-based neuro-linguistic programing (NLP) algorithms that combine basic keyword searching with rules to identify negations or context modifying instances and carry variable sensitivity, specificity, and lower negative predictive values (NPVs) and positive predictive values (PPVs) [2]. A low PPV suggests a poor performance by the algorithm to detect negations and context modifying instances so that sentences that should be excluded from the output search are ultimately included. However, it should be pointed out that PPV is affected by the overall prevalence of the condition of interest. With conditions of low prevalence, a significant proportion of false positives can be identified with anything but close to 100% specificity [2]. Ultimately, any of these algorithms should be tested using conditions of variable prevalence, to describe performance in light of disease characteristics. Recently, lack of standardization in reporting text mining algorithm performance has been described with a suggestion to particularly include data on sensitivity and PPV particularly in these studies [2].

Computer-assisted methodologies to extract information of interest from free-text EMRs have been used to report disease prevalence and for syndromic surveillance in veterinary medicine [12,13] with similar performance to algorithms utilized for processing human EMRs [13]. Although EMR-based research is more limited in veterinary medicine, a method using commercially available software (WordStat, Provalis Research) has been validated showing great potential in EMR-based research, whether veterinary or medical. The advantage of this software is a user-friendly interface that requires minimal training for the operator, and therefore, would be suitable for use by operators without a background in bioinformatics. The software provides the opportunity to adopt user-defined rules to identify negation terms and improve the specificity and PPV [13]. Despite obvious anatomical and pathophysiological differences between human and veterinary patients, EMR management systems share similar structure, goals, and modalities, and the use of text-mining procedures to search data of interest stored as free-text in veterinary EMR databases would also be applicable to human medical EMR databases.

The aim of this study was to describe in detail the text mining process using WordStat and validate its use against manual analysis performed separately by an experienced clinician by reading and interpreting the same data.

## Methods

### Data Used for Validation

The same dataset (validation dataset) was used for computer-assisted and manual analyses. This was created from a random selection of lifelong clinical records that were extracted using statistical software (R v3.0.2) from a random sample of equine patients from a greater dataset of 2,653,698 rows of data (cases), including 538,193 unique words used a total of 52,039,966 times, from 141,543 patients, obtained from 10 first opinion equine veterinary practices in the United Kingdom, and stored in the .csv format. Each case identifies the content pertinent to that patient on a single row of the .csv file. Each row of data had been generated at each visit but multiple rows could have been generated on that same visit, for example, one row could have been reporting clinical findings, another drug dispensed, and another some management notes. One patient would have contained from a single to several rows of data.

Validation was performed on 4 categories, including three conditions and one for drug use. Colic is a condition of middle-high prevalence in the horse population. Right dorsal colitis and renal failure were included as conditions with a low prevalence in first opinion settings. Finally, a fourth category of non-steroidal anti-inflammatory drug (NSAID) was included to validate the mining process to identify medication prescribing.

### Validation Process Design

This study compares the described computer-assisted classification process with that of manual analysis, which is included as the gold standard method of interpretation and classification of free-text clinical notes. The study was completed sequentially in 3 main steps. The first step consisted in the computer-assisted classification. The second step consisted in manual classification. The third step was the comparison of the results from each classification technique. Sensitivity, specificity, PPVs, NPVs, and *F* values of the computer-assisted process compared with the manual process were subsequently calculated by looking at where discrepancies were present between the two classification processes.

#### Computer-Assisted Classification

##### Inclusion Dictionary

The first step of the computer-assisted classification consisted in the manual evaluation of the list of all words included in the dataset that had been created by the function “Frequencies” in WordStat and then exported into a spreadsheet. Any word that might have identified any of the above categories was included in the inclusion dictionary relevant for that category. This included terms spelled correctly, spelled incorrectly but judged to likely refer to one of these categories, or abbreviated. Following the creation of the inclusion dictionaries, cases containing words contained in the categorization dictionary were extracted via the “keyword-in-context” function.

The result of the search for each of the categorization dictionaries consisted in a spreadsheet with 5 columns: one for data row number, one for the text preceding the word or combination of words identified by the search, one for the word or combination of words itself, one for the text after the word or combination of words, and one for the patient’s anonymous identification. The spreadsheet was saved as a .csv for subsequent evaluation.

##### Exclusion Dictionary

The output of the search obtained from the inclusion dictionary was subsequently evaluated manually to identify word combinations identifying false positive cases. Each exclusion dictionary was created with combinations of words identifying false positive cases. Once a comprehensive exclusion dictionary had been created, cases containing combinations of words in the exclusion dictionary were identified and were exported as a .csv file.

Both search results from inclusion and exclusion characterization dictionaries were imported in R v3.0.2 so that the rows containing false positive data identified by the exclusion dictionary could be removed from the search results of the inclusion dictionary.

The dataset “Results” included all the cases from the original dataset that included words in the inclusion dictionary but that also excluded the cases containing combinations of words specified in the exclusion dictionary.

##### Reinclusion Dictionary

The output search of the exclusion dictionary was also evaluated manually to identify whether it included false negative cases. Combinations of words uniquely identifying these false negatives were included in the reinclusion dictionary and reincluded in the Results dataset using R v3.0.2.

**Figure 1:**
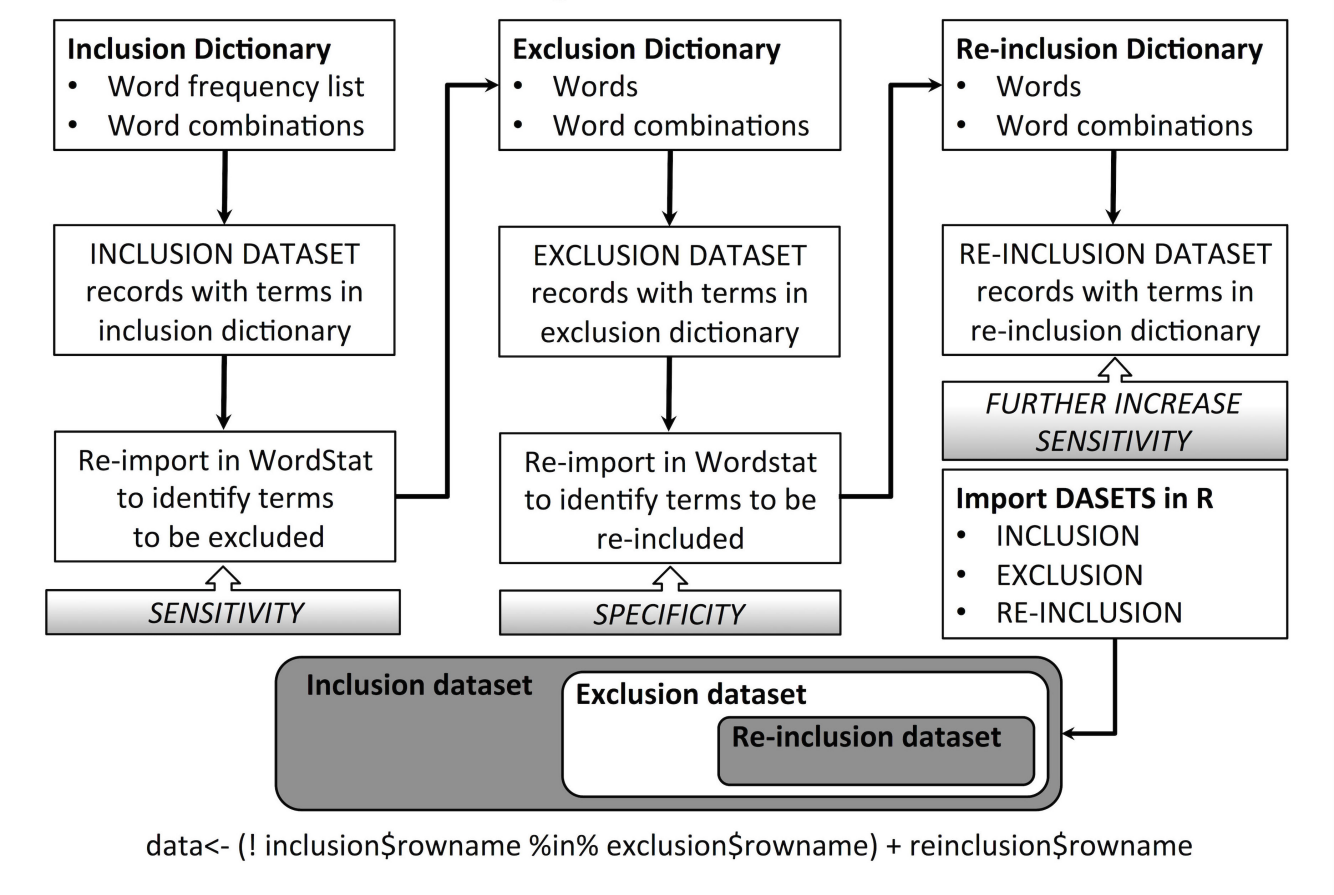
Flowchart summary of the text mining process adopted in the study. The lower portion of the picture summarizes how the final dataset (dark gray) resulted from the subtraction of the exclusion dataset from the inclusion dataset and the final addition of reinclusion dataset obtained from the exclusion dataset.

The Results datasets obtained for each of the 4 categories investigated by computer-assisted classification consisted in a subset of the validation dataset including the records (with the original row-number) identifying one of the four categories sought. The text-mining process is summarized in Figure 1.

#### Manual Classification

The validation dataset was classified manually independently from computer-assisted classification. Manual classification was performed entirely in MS Excel where a column for each of the 4 categories investigated was added to the spreadsheet containing the original data. Each row of data was manually tagged according to one of the categories: “NSAIDs,” “colic,” “renal failure,” and “right dorsal colitis.” Where a row of data identified more than one category, multiple tags were applied accordingly. Manual classification was performed by a single, experienced equine clinician (holding a degree in veterinary medicine as well as specialist qualification in equine internal medicine), and tag allocation was based on the interpretation of each row of data in the dataset. The dataset was manually evaluated once.

Manual classification results consisted in the .csv validation dataset with an added column for each of the categories sought and a tag in the rows identifying the relevant category.

#### Comparison between Computer-Assisted and Manual Classifications

The search output for each of the 4 characterization dictionaries and the validation dataset, including the column with the manual classification, were imported in R v3.0.2. Computer-assisted and manual classifications were compared and any discrepancy recorded and subsequently reevaluated manually to investigate the source of the disagreement. A two-by-two contingency table was produced for each dictionary and sensitivity, specificity, PPVs, NPVs, and *F*-measure were calculated.

The study was performed with the approval of the Research Ethics Committee of the School of Veterinary Medicine of the College of Medical, Veterinary and Life Sciences at the University of Glasgow.

## Results

### Data Used for Validation

The clinical records of 335 patients, including 17,561 cases from the main dataset, were obtained representing 0.2% of all animals and 0.7% of all data. The average number of cases per animal was 52.3 (median 14; range 1-1031; 1^st^quartile: 5; 3^rd^quartile: 44.2). Free-text data included 16,882 unique words used a total of 538,193 times. The data included columns for anonymous patient ID, date of data entry, and a column for free-text clinical notes, which included a mixture of notes entered by the clinician as well as text, including information of drug prescription and sales. There was no standardized diagnostic coding or fixed vocabulary in the dataset.

#### Computer-Assisted Classification

##### Inclusion Dictionary

The inclusion dictionary for “NSAIDs” included 53 words, 57 words for “colic,” 13 words for “renal failure,” and six words for “right dorsal colitis.” Words in the NSAID inclusion dictionary were present 1562 times in 1181 cases for NSAIDs, 356 times in 291 cases for colic, 23 times in 23 cases for renal failure, and 7 times in 7 cases for right dorsal colitis.

##### Exclusion Dictionary

The exclusion dictionary for “NSAIDs” included 4 combinations of words, 131 combinations of words for “colic,” 4 combinations of words for “renal failure,” and none for “right dorsal colitis.” The combinations of words in the NSAIDs exclusion dictionary were present 125 times in 112 cases, 63 times in 57 cases for colic, twice in one case for renal failure, and none for right dorsal colitis.

##### Reinclusion Dictionary

The reinclusion dictionary for “NSAIDs” included 4 combinations of words, 5 combinations of words for “colic,” and none for “renal failure” and “right dorsal colitis.” Following data extraction of the total of 17,561 cases in the validation dataset, combinations of words in the NSAIDs reinclusion dictionary were present 79 times in 78 cases and 5 times in 5 cases for colic. No term was present for both renal failure and right dorsal colitis.

**Figure 2:**
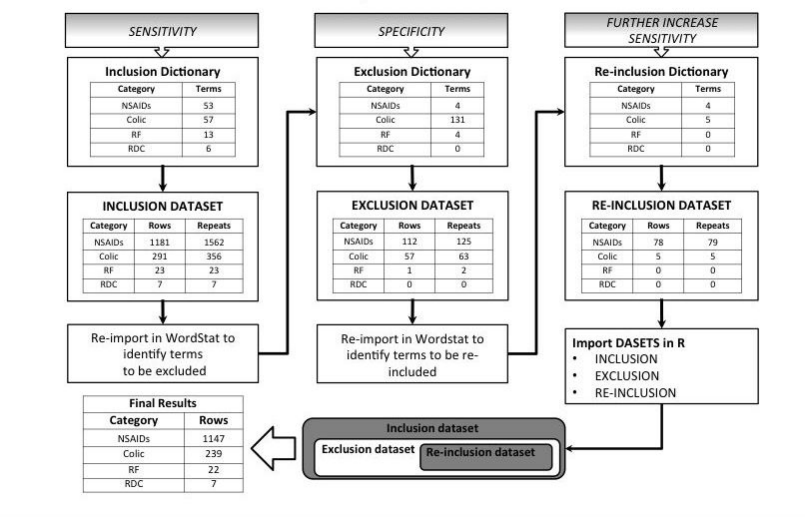
Flowchart summary of data flow of the computer-assisted text mining process for the validation dataset. The columns include either the number of terms (each term is either a word or a combination of words) in each dictionary, cases, or number of times (“repeats”) terms in the relevant dictionary are identified in the dataset.

Computer-assisted classification was performed in seconds, though the process of dictionary creation was lengthy and required approximately 30 h in total for a dataset of this size. Computer-assisted classification resulted in the identification of 1147 cases for NSAIDs, 239 cases for colic, 22 cases for renal failure, and 7 cases for right dorsal colitis. Data flow for the computer-assisted classification process is summarized in Figure 2.

#### Manual Classification

Manual classification of the validation dataset of 17,561 cases identified 1130 cases where NSAID prescribing was identified, 226 cases for colic, 22 cases for renal failure, and 7 cases for right dorsal colitis. Manual classification was completed in 80 h performed over a period of 10 days.

#### Comparison Between Computer-Assisted and Manual Classifications

The results of the comparison of computer-assisted and manual analysis including determination of sensitivity, specificity, PPV, NPV, and *F* value are summarized in Table 1. Overall, there was excellent agreement between computer-assisted and manual analysis.

Computer-assisted classification correctly identified 19 cases that were erroneously classified as negative by manual classification. These were cases where NSAIDs had in fact been prescribed but were missed while reading through the dataset once. Two further cases were false negative cases incorrectly classified by the computer-assisted process but correctly identified by manual processing. These referred to a hypothetical or future use of the drug. For example, one sentence commented that “c/s + NSAID not option (as low TP and previous laminitis),” and the other wrote “NSAID in future.” Similarly, there were 13 cases classified as “colic” by the computer-assisted classification but classified not as colic by the manual process. These cases referred to instances where it was not clear whether the case was indeed a colic, where the investigation for colic have been performed but the results are not consistent with colic. For example, “neighbours reported horses colicking now seems fine HR etc normal,” the colic was unconfirmed or very short lived so it is debatable whether this really had consisted in a colic case. Another example such as “soft F+ present in rectum.no impaction palpable” suggests that there findings are unremarkable, yet if trans-rectal palpation was performed and noting the lack of an impaction was required, then the horse might indeed have exhibited signs of colic, so again, whether this case should be classified as “colic” is open to debate. The dataset included 22 cases referring to renal failure and 7 cases referring to right dorsal colitis and were all correctly identified by both methodologies.

**Table 1:** Sensitivity, specificity, and positive and negative predictive values (PPV and NPV, respectively) of computer-assisted analysis compared with manual analysis reported (values reported as per cent values). Rows are conditions identified by the software and columns correspond to manual classification.

Category		Manual^a^		Sensitivity	Specificity	PPV^b^	NPV^c^	*F*
	C-A^d^	+	−					
NSAIDs^e^								
	+	1128	19	99.8	99.9	98.3	100	99.0
	−	2	16410					
Colic								
	+	226	13	100	99.9	94.6	100	100
	−	0	17322					
RF^f^								
	+	22	0	100	100	100	100	100
	−	0	17539					
RDC^g^								
	+	7	0	100	100	100	100	100
	−	0	17554					

^a^+/− in the “manual” column identifies the number of positive and negative terms classified manually in each category (Colic, nonsteroidal antiinflammatory drugs [NSAIDs], renal failure [RF], and right dorsal colitis [RDC]).

^b^PPV: positive predictive values.

^c^NVP: negative predictive values.

^d^The +/− in the “C-A” column identifies the number of positive and negative terms classified with the computer-assisted method for each category.

^e^NSAIDs: nonsteroidal antiinflammatory drugs.

^f^RF: renal failure.

^g^RDC: right dorsal colitis.

## Discussion

### Principal Findings

The findings of this study show that the methodology described yields results very similar to manual analysis. This methodology is suitable for studies using large free-text EMRs that require the highest possible sensitivity, specificity, PPVs, and NVPs. The technical time required to automatically mine the information of interest from the dataset is negligible (after creation of the relevant dictionaries) in comparison with that of manual analysis. A few seconds are required with the computer-assisted process, depending on machine power, compared with approximately 80 h for the manual analysis for a dataset of 17,561 cases. The study reported here used less than 1% of all available records, and this differential in time required will obviously only get bigger as the dataset increases in size. It is important to point out that a variable amount of time is necessary initially to create adequately comprehensive dictionaries, and this is dependent on the type of dictionary that is being created. Creation of dictionaries identifying a specific diagnosis or to identify drug prescription is faster as the terminology used by clinicians is generally limited and specific. On the other hand, creation of dictionaries that identify a syndrome or a list of generic clinical signs or presenting complaints is highly dependent on the multitude of possible colloquial descriptions that might identify that condition. Creation of exclusion and inclusion dictionaries also requires a degree of manual evaluation of the search output, which would be more time consuming for larger datasets, but not in a linear manner. For example, the number of unique words in the validation dataset was proportionally 10 times greater than in the original dataset. In this study it was noticed that in most cases a relatively small number of word combinations identified the vast majority of false positive and false negative cases, which made exclusion and reinclusion dictionary definition much faster. Furthermore, alphabetic ordering of records by keyword and in context evaluation were performed rapidly as clinicians have the tendency to adopt the same combination of words to describe similar clinical scenarios, hence, reducing grammatical variability and speeding up the analysis process. A novelty of this methodology is in the use of a reinclusion dictionary, which promotes a further increase in the overall method specificity without compromising in sensitivity. This method is therefore suitable for studies where optimal identification of cases is required. A further advantage is the ease-of-use of the software that makes this method suitable to operators without any prior background in bioinformatics.

The relatively high number of false positive cases detected by the computer-assisted process consisted of truly positive cases that had been missed during manual analysis. The vast majority of discrepancies between computer-assisted and manual classification was for cases classified as false positives by the computer-assisted process. On reevaluation, these were in fact found to be correctly classified and had been missed by manual evaluation. This finding highlights that a single-operator manual analysis chosen as a gold standard method for free-text analysis is not perfect. Repeating the manual process by the same operator and second operator would have helped to evaluate intra- and inter-operator variability of manual process. Combining the results of 2 operators could have improved the outcome of manual analysis. However, the process of manual analysis was very time consuming, and it was expected that one operator was sufficient for the purpose of the study to compare the computer-assisted and manual processes.

The EMRs used for the study were obtained from veterinary practice. However, despite the anatomical and physiological differences between veterinary and human patients, the terminology used to describe clinical scenarios is very similar if not identical. The slight differences in terminology would be easily addressed during the dictionary definition process, therefore, the method described here would be suitable for use in case-detection research of human patient free-text data.

### Limitations of This Process

A limitation of the described method is the component of manual data checking to compile exclusion and reinclusion dictionaries. This requires a considerable effort by the operator, eased by the software, but that remains somewhat proportional to the size of the dataset to be analyzed. When compared with more conventional techniques, using computer-assisted rule-based case definition this time and effort is compensated for by the overall improved performance. A further limitation of this study is the use of a single observer for the manual analysis. Comparing the manual analysis performed independently by 2 operators would have provided a mean of validation of the manual analytic process. Similarly, this study compares manual analysis with the combined computer-assisted process, and the excellent agreement between the 2 methods is acceptable to demonstrate that both methods worked equally well. Finally, since the dictionaries are created from the data, this methodology is suited mostly for retrospective evaluation of EMRs, and if new data is being analyzed, then dictionaries should be updated on the new data to ensure maximal specificity and sensitivity.

A limitation of the validation process described in this study lays in the fact that some patients included in the validation dataset had contributed with a different number of cases. Since this methodology aims at identifying the cases referring to the condition of interest and not the patient affected, ideally the dataset should have included a random selection of rows from the original dataset. However, including a random selection of cases of similar size would have likely resulted in only few or no cases containing text referring to the conditions being investigated. Alternatively, a dataset created from a random selection of cases including an equally large number of cases referring to these conditions would have been too large to be evaluated manually. The decision to include data from a selected number of patients was performed to evaluate this methodology over a wider array of lexical variation.

A further limitation of using WordStat may lie in the cost associated with purchase of the software. In early 2017, the software can be purchased by academics for 695USD (1995USD for governmental organizations and 3795USD for commercial companies), which may at first appear expensive in light of the other software available freely, but the cost may be outweighed by the high sensitivity and specificity offered with this procedure. Whether the methodology of using word frequency list, and inclusion, exclusion, and re-inclusion dictionaries could also adapt to other open source software, should be evaluated in future studies.

### Comparison With Prior Work

Excellent specificity and sensitivity was expected as each dictionary was created using a list of words obtained from the dataset and included misspelled and abbreviated terms associated with the category. Considerable effort was required to create a comprehensive inclusion dictionary accounting for all misspellings and abbreviations but was essential to maximize sensitivity of the analytic process. The reinclusion dictionary also further contributed to improving sensitivity where necessary. Although browsing through the word list was a time consuming but pivotal step of the analytic process, it also means that the dictionary is very dataset-specific, and if new data, especially from different clinical practices or veterinarians, is added, then the dictionary should be updated to include new terms. All parameters used to evaluate the performance of the analysis process were superior to most of the current techniques reported in a recent systematic review on case detection from EMRs [2]. The improved performance of the current method is the result of an increased work-load of the operator as the rule-based portion of the analysis to identify negations and context modifying instances is performed somewhat manually. The study by Anholt and colleagues (2014) validated the use of the same software package using rule-based case definition to identify negations and context modifying instances and reported an inferior sensitivity comparable with that of other NLP algorithms [2]. However, that study was aimed at syndromic surveillance where specificity was prioritized over sensitivity in order to minimize false positive rates. This highlights how study design dictates which of these text mining methodologies is more suitable. Future studies aimed at describing disease prevalence or risk factor analysis may have stricter requirements of test performance, and the increased effort of the current method could be justified by the increased reliability of the results produced.

### Conclusions

In conclusion, the computer-assisted process is significantly faster once inclusion, exclusion, and reinclusion classification dictionaries are prepared on a dataset of this size while preserving performance at least as good as manual analysis. As all words present in the dataset are used, sensitivity does not appear to be an issue for this method. In terms of optimization of specificity and sensitivity, the use of exclusion and reinclusion dictionaries is useful in situations where there are many false positive and false negative cases. This is achieved simply by evaluating the output search of the inclusion dictionary to identify any significant proportion of erroneous classifications. False positive and negative cases appeared proportionally more common when trying to identify general syndromes, such as colic, but less common when focusing on specific diagnosis or when looking at drug administration. Future area of research should aim at improving the dictionary definition process to make the process more versatile and adaptable to new data. As technologies improve, this method will probably become obsolete as sensitivities, specificities, PPVs, and NPVs of fully computer-assisted processes, whether rule-based or probabilistic, are likely to improve in the future. These processes should ultimately reduce the operator’s effort for dictionary creation and be adaptable to new data.

## Abbreviations

EMR: electronic medical record
NPV: negative predictive value
NSAID: non-steroidal anti-inflammatory drug
PPV: positive predictive value
